# Consolidated Bioprocess for Bioethanol Production from Raw Flour of *Brosimum alicastrum* Seeds Using the Native Strain of *Trametes hirsuta* Bm-2

**DOI:** 10.3390/microorganisms7110483

**Published:** 2019-10-23

**Authors:** Edgar Olguin-Maciel, Alfonso Larqué-Saavedra, Patricia E. Lappe-Oliveras, Luis F. Barahona-Pérez, Liliana Alzate-Gaviria, Rubí Chablé-Villacis, Jorge Domínguez-Maldonado, Daniella Pacheco-Catalán, Hector A. Ruíz, Raúl Tapia-Tussell

**Affiliations:** 1 Renewable Energy Department, Yucatan Center for Scientific Research, Merida 97302, Mexico; edgar.olguin@cicy.mx (E.O.-M.); barahona@cicy.mx (L.F.B.-P.); lag@cicy.mx (L.A.-G.); rubi.chable@cicy.mx (R.C.-V.); joe2@cicy.mx (J.D.-M.); dpacheco@cicy.mx (D.P.-C.); 2 Natural Resource Department, Yucatan Center for Scientific Research, Merida 97205, Mexico; larque@cicy.mx; 3 Mycology Laboratory, Biology Institute, National Autonomous University of Mexico, Mexico 04510, Mexico; lappe@ib.unam.mx; 4 Biorefinery Group, Food Research Department, Faculty of Chemistry Sciences, Autonomous University of Coahuila, Saltillo 25280, Mexico; hector_ruiz_leza@uadec.edu.mx

**Keywords:** consolidated bioprocess, biofuels, starch, α-amylase, white rot fungi

## Abstract

Consolidated bioprocessing (CBP), which integrates biological pretreatment, enzyme production, saccharification, and fermentation, is a promising operational strategy for cost-effective ethanol production from biomass. In this study, the use of a native strain of *Trametes hirsuta* (Bm-2) was evaluated for bioethanol production from *Brosimum alicastrum* in a CBP. The raw seed flour obtained from the ramon tree contained 61% of starch, indicating its potential as a raw material for bioethanol production. Quantitative assays revealed that the Bm-2 strain produced the amylase enzyme with activity of 193.85 U/mL. The Bm-2 strain showed high tolerance to ethanol stress and was capable of directly producing ethanol from raw flour at a concentration of 13 g/L, with a production yield of 123.4 mL/kg flour. This study demonstrates the potential of *T. hirsuta* Bm-2 for starch-based ethanol production in a consolidated bioprocess to be implemented in the biofuel industry. The residual biomass after fermentation showed an average protein content of 22.5%, suggesting that it could also be considered as a valuable biorefinery co-product for animal feeding.

## 1. Introduction

The production of bioethanol for the transport sector is considered an important and necessary measure for reducing the dependence of modern society on fossil energy. Nowadays, fossil fuels cover 90% of the growing demand for energy. Unfortunately, with the current rate of fossil fuel consumption, it is expected that reserves will be exhausted within the next 40 to 50 years. More importantly, the burning of fossil fuels contributes to global warming due to greenhouse gas emissions, causing climate change, a rise in sea levels, loss of biodiversity, and urban pollution [1,2,3]. Among the alternative fuels options, bioethanol offers an immediate solution because it does not require to modify the current transport infrastructure. The use of bioethanol can reduce oil consumption as well as environmental pollution. Production and consumption of ethanol have been promoted through subsidies, mandates, and financing for research, and in fact, more than 64 countries have participated in various programs to use bioethanol as their main source of fuel. Ethanol blends vary depending on the country, containing from as low as 5% (E5) to 100% bioethanol (E100) [4,5,6,7].

The growing global demand for bioethanol requires the use of alternative sources of raw materials to complement sugar cane and cornstarch, which are the main raw materials used to produce it. Starch crops are widely used for bioethanol production because of their worldwide availability, easy conversion, and high ethanol yield. These raw materials include cereals (60%–80% starch), tubers and roots (60%–90%), legumes (25%–50%), and green and immature fruits (up to 70% starch) [2].

Conventionally, ethanol production from starch consists of several stages. Starch is subjected to a gelatinization process followed by a liquefaction step where starch is converted to dextrins and smaller molecules by the action of bacterial thermostable amylases at high temperatures (95–105 °C) and pH values between 6 and 6.5. This step is followed by saccharification. The liquefied starch is cooled, pH is adjusted to 4–4.5, temperatures to 60–65 °C, and a fungal glucoamylase is added to hydrolyze the oligosaccharides to glucose. The liquefaction and saccharification stages represent about 40%–50% of the total energy used during starch-based ethanol production [8,9,10].

Owing to this technical complexity and the economic implications of this approach, other biological alternatives have been investigated, such as simultaneous saccharification and fermentation (SSF) and consolidated bioprocessing (CBP). The latter is a promising strategy for effective ethanol production, since it employs only one type of microorganism that is capable of both producing the enzymes to hydrolyze the biomass and converting sugars into ethanol [11,12]. This strategy has the potential of lowering the cost and enhancing the efficiency of bioethanol production, as compared to methods based on separate hydrolysis and fermentation. To achieve a cost-effective conversion of starch to bioethanol it is also worth considering the valorization of the by-products from this process. They are a rich source of protein, fiber, fat, vitamins, and minerals that can be used (wet or dried) as a component of livestock forage [11,12,13,14]. However, a large-scale techno-economic analysis of consolidated bioprocessing has been scarcely reported in the literature [15].

CBP requires the selection of suitable microorganisms, and bacteria and yeasts such as *Clostridium thermocellum* and *Scheffersomyces shehatae* [14,16] have been the primary candidates for CBP research [17,18]. Fungi have not been widely proposed as CBP microorganisms, but several natural filamentous fungi have also been reported as directly converting cellulose to ethanol, including the genera *Aspergillus*, *Rhisopus*, *Monilia*, *Neurospora*, *Fusarium*, and *Trichoderma* [19]. Basidiomycetes, also known as wood-rotting fungi, are considered primary agents of plant litter decomposition in terrestrial ecosystems. Furthermore, some basidiomycetes produce alcohol dehydrogenases, thus allowing the production of wine using a mushroom [20]. In this regard, some wood-decaying basidiomycete fungi were reported to produce ethanol from hexoses, pentose, starch, wheat bran, and rice straw. These results indicate that white-rot fungi are good candidates for bioethanol production from woody biomass [19,21,22,23,24].

In this work, the use of the Bm-2 strain of *Trametes hirsuta* is proposed in a CBP for bioethanol production using flour made from ramon or breadnut tree (*Brosimum alicastrum*) seeds as a substrate. Ramon is a forest tree native to Mesoamerica and the Caribbean and is widely distributed in México, In the Yucatán Peninsula, México, breadnut is found virtually in every backyard or garden of the Mayan families, with a production of 95.5 kg per tree per year. Nowadays, this seed is rarely used for human consumption among the population of the Yucatan Peninsula. This species has suitable characteristics for producing bioethanol, such as high productivity, tolerance to drought, and a starch content of 61% [25,26,27]. In a previous work, Olguin-Maciel et al. [28] obtained 214 L of ethanol per ton of flour using separate hydrolysis and fermentation (SHF) and *Candida tropicalis* in the fermentation process. 

The aim of this works was to develop a CBP using ramon seed flour as raw material and *T. hirsuta* Bm-2 strain as the microorganism for the fermentation process. In addition, the strain’s capacity to produce hydrolytic enzymes and its tolerance to ethanol were studied.

## 2. Materials and Methods 

### 2.1. Raw Material

*B. alicastrum* seeds were collected from different locations in the State of Yucatan, Mexico. The seeds were dried in a convection oven (Binder, Fed model 115^®^, Tuttlingen, Germany) at 70 °C for 72 h, after which the seed coats were mechanically separated, and the clean seeds were stored in a desiccator until milling. The ramon flour (RF) used in this work contained 75% of carbohydrates, of which 61% was starch, and 12.24% was total proteins [28].

### 2.2. Fungal Strain and Cultivation Conditions

The *T. hirsuta* Bm-2 strain (GQ280373) was isolated from decaying wood in Yucatan, Mexico, and its molecular identification was based on the analysis of Internal Trancribed Spacers of nuclear ribosomalDNA (ITS regions) [29]. The strain was maintained by periodic subculturing on 14% RF plates. A mycelia suspension of *T. hirsuta* Bm-2 was obtained by inoculating 2 × 1 cm-diameter dishes of mycelium after 5 days growth in a 250 mL Erlenmeyer flask containing 100 mL of yeast extract and starch (YS) liquid medium, pH 5, which consisted of (g/L): yeast extract, 4.0; MgSO_4_·7H_2_O, 0.5; K_2_HPO_4_, 1.0; the starch was replaced with RF (15.0 g/L). The flask was incubated at 32 ± 2 °C and 150 rpm for 6 days. During this period, α-amylase and laccase activities were evaluated every 24 h. The biomass obtained was homogenized with the T18 digital ULTRA-TURRAX^®^ by IKA^®^ (Staufen, Germany). The resulting suspension was used as inoculum in the CBP.

### 2.3. Enzyme Assay

#### 2.3.1. Analysis of α-amylase Activity

To study the ability of the Bm-2 strain to hydrolyze soluble potato starch, (Sigma Aldrich, St Luis, MO, USA), Petri plates with YS solid medium were inoculated with a 1 cm-diameter dish of mycelium grown for 5 days, incubated at 32 ± 2 °C, and evaluated (two different plates) every 48 h for 6 days. The plates were flooded with Lugol’s iodine solution (Sigma Aldrich) to monitor the production of a starch degradation halos after iodine staining [30]. A pale-yellow zone around the mycelium indicated starch degrading activity.

The activity of α-amylase was determined by a modified method, following Ahmed et al. [31]. The assay mixture consisted of 0.5 mL soluble starch as substrate (1% in 0.1 M sodium acetate buffer pH 5.0) and 0.5 mL of crude extract. After incubation for 20 min at 40 °C, the reaction was stopped by cooling the sample to 4 °C. After cooling, starch hydrolysis was determined through glucose release by the Miller DNS method [32]. The amount of enzyme production was expressed as U/mL. A unit of enzyme activity was defined as the amount of enzyme that released 1 µg of reducing sugar as glucose standard per minute under assay conditions.

#### 2.3.2. Analysis of Laccase Activity

Petri dishes with malt extract (2%) and agar (2%) containing 5 mM 2,2’-azino-bis (3-ethylbenzthiazoline-6-sulphonic acid (ABTS) were inoculated with a 1 cm-diameter dish of mycelium grown for 5 days and incubated at 32 ± 2 °C for 4 days. The formation of a dark-green halo on the plates indicated a positive extracellular laccase secretion [29].

Laccase activity in cell-free filtrates was measured at 40 °C using ABTS. The assay mixture contained 1 M sodium acetate buffer (pH 4.5) and 0.5 mM ABTS in a total volume of 1 mL. The oxidation of ABTS was measured by the increase in absorbance at 420 nm, as described by Johannes and Majcherczyk [33]. One enzyme unit (U) is defined as the amount of enzyme required to oxidize 1 µmol of ABTS per min under assay conditions. The amount of enzyme production was expressed as U/mL.

### 2.4. Tolerance to Ethanol

Tolerance to ethanol was evaluated by inoculating a 1 cm-diameter dish of mycelium grown for 5 days on Petri dishes of yeast extract–malt extract agar medium (YMA), which consists of (g/L): glucose, 18.0; yeast extract, 3.0; malt extract, 3.0; peptone, 5.0, and agar 20.0. Different concentrations of absolute ethanol were added in increments of 1% (6%–14%, *v*/*v*), and the plates were incubated at 32 ± 2 °C. The autoclaved medium was cooled to 45–50 °C, and warm ethanol was added immediately before pouring. The plates were sealed with parafilm soon after setting of the agar and again after inoculation. The evaluation was done 10 days after inoculation [34].

### 2.5. Consolidated Bioprocess Conditions

Experiments were performed in 250 mL Erlenmeyer flasks capped with cotton plugs, which allowed the transfer of oxygen during the CBP. A suspension of RF (100 mL, 14% *w*/*v*) in distilled water was prepared and autoclaved at 121 °C for 15 min (Yamato Sterilizer model SM510, Tokyo, Japan). The samples were cooled to room temperature, inoculated using 1 mL of inoculum prepared as described in Section 2.2 above, and then incubated at 32 ± 2 °C for 12 days in stationary conditions in a drying chamber (Binder, Fed model 115, Tuttlingen, Germany). Samples of 1.5 mL of the supernatant were then taken for analysis (glucose liberation, ethanol production, and assessment of enzyme activity) at various times (0, 48, 96, 144, 192, 240, and 288 h). 

At the end of the CBP, the fungal mycelium was removed from the flask and washed with distilled water for three times. The liquid fraction and the residual RF were separated by centrifugation at 8000 rpm for 10 min. The recovered liquid was stored at 4 °C for further analysis. Both the recovered mycelium and the residual RF were dried at 60 °C to a constant weight, ground, and sieved with a No. 40 mesh. The protein content was determined in the mycelium and in the residual RF [35], while starch was determined in the residual RF only [36]. All experiments were performed in triplicate.

### 2.6. Concentration of Ethanol and Glucose

All samples taken from CBP were filtered through a 0.2 µm sterile membrane filter and analyzed for glucose and ethanol content by HPLC (1260 Infinity II, Agilent, Santa Clara, CA, USA). Chromatographic separation was performed using a Metacarb 87H column 300 × 7.8 mm (Agilent) under the following conditions: mobile phase H_2_SO_4_ (0.005 M), flow rate 0.7 mL/min, and column temperature 60 °C. The system comprised a Jasco chromatograph 880-PU intelligent pump (Jasco, Tokyo, Japan) equipped with a Jasco 830-IR intelligent refraction index detector (Jasco) and a Jasco AS-2057 Plus intelligent autosampler (Jasco). The volume injected was 20 µL per sample. Sugars and ethanol concentrations were determined on the basis of the calibration curves of these pure compounds [37].

The fermentation efficiency was calculated from the theoretical ethanol yield using Equation (1) [38].

(1) $$\mathrm{Fermentation}\;\mathrm{efficiency}=\frac{\mathrm{Ethanol}\;\mathrm{produced}\;\left(\frac{g}{\mathrm{kg}}\mathrm{starch}\right)}{\mathrm{Theorical}\;\mathrm{yield}\;\left(\frac{g}{\mathrm{kg}}\mathrm{starch}\right)}\times 100$$

The theoretical yield of ethanol (567 gm/kg starch) was calculated using Equation (2) [38].

(2) Theoretical yield (g/kg starch)=(511a × 1.11b)

where:

a = theoretical yield (g) of ethanol from 1.0 kg glucose [39].
b = yield of glucose (kg) from 1.0 kg starch [39].

### 2.7. Physical-Chemical Characterization

Elemental analysis (C, H, N, and S) was carried out using a Thermo Scientific Elemental Analyzer Flash 2000 (Thermo Scientific, Waltham, MA, USA). The functional groups on the carbon surface were determined by Fourier transform infrared spectroscopy (FTIR) with a Bruker FT-IR Tensor II (Bruker, Ontario, ON, Canada). This analysis was carried out with the attenuated total reflection (ATR) accessory. Thermogravimetric analyses (TGA) of initial and final samples were carried out using a Perkin Elmer TGA 8000 (PerkinElmer, Walthman, MA, USA). A TG curve was obtained under a nitrogen atmosphere with a flow rate of 10 mL/min and a heating rate of 10 °C/min from 50 to 700 °C with a sample mass of 10 mg in a platinum pan. The mass loss rate in the derivative form (derivative thermogravimetry, DTG) was also calculated. 

Morphological characterization was also carried out with a scanning electron microscope (SEM, model JSM-6360LV, JEOL, Tokyo, Japan). Dry flour samples were mounted on a metallic stub using double-sided adhesive tape coated with a 15 nm gold layer and observed at 20 kV. After bioprocessing, the samples were fixed in 2.5% glutaraldehyde in a 0.02 M sodium phosphate buffer (pH 7.1) for 48 h (24 h at room temperature and 24 h at 4 °C). This was followed by six 30 min washes in a 0.02 M sodium phosphate buffer at 4 °C and then dehydratation in graded ethanol series (30%, 40%, 50%, 60%, 70%, 85%, 95%, and 100% *v*/*v*, twice for 30 min). For SEM analysis, the samples were critical-point dried in CO_2_. After fixation and dehydration, the samples were processed in the same way as the initial flour [40].

The concentration of phenols was determined in triplicate by the Folin–Ciocalteu method [41], at 740 nm. The phenolic content of the samples was expressed as mg gallic acid equivalents.

## 3. Results and Discussion

### 3.1. Enzyme Activity

In a first approach, the ability of *T. hirsuta* Bm-2 to produce amylase, the main enzyme used in the hydrolysis of starch, was evaluated. Then, the production of laccase enzyme was studied, as *Basidiomycota* is recognized as the most relevant phylum regarding the secretion of laccases [42].

**Figure 1:**
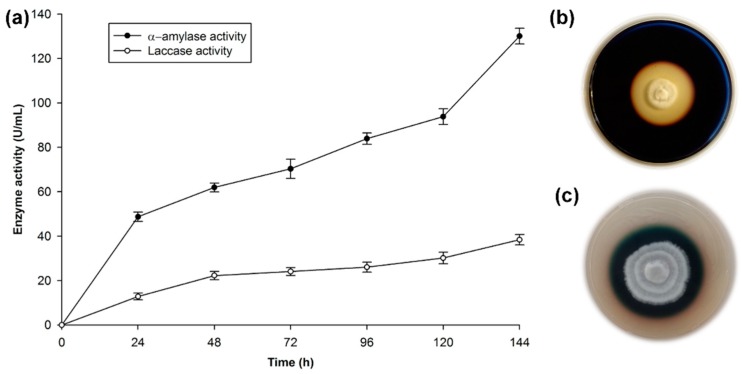
Test of the enzyme activity of the *Trametes hirsuta* Bm-2 strain. (**a**) Quantitative activity of laccase and α-amylase enzymes, (**b**) screening for amylolitic activity in plates incubated at 32 °C for 144 h, (**c**) screening for laccase activity in plates incubated at 32 °C for 96 h. The results are represented as the mean ± standard deviation of three parallel measurements (*n* = 3).

The amylase test in Petri plates with YSA medium resulted in clear halos around the mycelial growth of *T. hirsuta* Bm-2, after 6 days of cultivation (Figure 1b). A 38 mm diameter hydrolysis zone around the mycelium was measured; this was due to the extracellular production of amylase. The amylase enzyme is produced by many microorganisms, and the *Bacillus* genus is considered to be the main source of this enzyme for commercial purposes. In the case of fungi, the reports are limited to a few species. The fungal sources are limited to terrestrial isolates, mostly *Aspergillus* and *Penicilium* [43]. In the case of *Basidiomycete* species, there are few reports of the production of this type of amylolitic enzymes, because these species are mainly used for their recognized capacity for ligninolitic enzyme production. However, the production of enzymes is influenced by the components of the medium, and amylase is induced in the presence of starch or its hydrolytic products such as dextrins and maltose [44,45], which are present in RF.

Figure 1c shows the characteristic dark-green halo that results from the oxidation of the ABTS indicator. The coloration around the mycelial growth indicates extracellular laccase activity in accordance with the work done on this strain [29,41]. *T. hirsuta* belongs to a small group of Basidiomycetes, known as white-rot fungi, which possess the unique ability to break down lignocellulosic biomass. This process can be accomplished through the production of two enzymatic systems: (1) A hydrolytic system, which produces cellulases and hemicellulases that degrade polysaccharides; and (2) An oxidative ligninolytic system, which degrades lignin and opens the phenyl rings. Lignin peroxidase, manganese peroxidase, and laccase are the key enzymes [46].

The profiles of extracellular enzymatic activities during cultivation of *T. hirsuta* Bm-2 are shown in Figure 1a.

The production of α-amylase reached its maximal level on the sixth day of growth, with approximately 135 U/mL of activity. This agrees with reference [47], who mentioned that amylase from a fungal source is normally produced after 3–7 days of incubation. There are a few reports of amylase production by Basidiomycetes, e.g., the amilolytic capacity of *T. hirsuta* has been qualitatively determined [21,48], as well as an enzymatic activity of 267 U/g for amylase in the Basidiomycete *Ganoderma lucidum* [49]. However, comparisons of the α-amylase activity level are very difficult due to the different methodologies used to determine the enzyme activity, the different manners of reporting the enzyme concentration, and the different ways of defining the units used to measure enzyme activity [44].

The ability of *T. hirsuta* to produce α-amylase extracellularly can contribute to establishing CBP production of bioethanol from starch material. It has the advantage of reducing the production costs with respect to the use of commercial enzymes, such as the energy costs, since these enzymes show their greatest activity in the same temperature range as that optimal for the growth of *T. hirsuta*, which produces them, in this case 32 ± 2 °C.

Laccase activity was 40 U/mL, as reported previously [29] for the same strain. This value is low, considering the recognized ability of this type of fungus to produce ligninolitic enzymes, as mentioned above. However, it should be pointed out that the medium used to prepare the inoculum in this study was starch-rich and induced the production of amylase, while for laccase induction in Basidiomycetes, aromatic compounds such as phenols associated with lignin are used, as mentioned in previous studies. In the presence of compounds such as ferulic acid, vanilline, guaiacol, and wheat bran, Tapia-Tussell et al. [41] and Zapata-Castillo et al. [50] obtained high lacasse activities of 2543.7 and 2496 U/mL, respectively. When they used low concentrations of these inductors, they reported low laccase activity (234.7 and 178 U/mL, respectively). In this study, the low laccase activity was due to the low concentrations of free phenols (2.90 mg/g of RF). 

### 3.2. Tolerance to Ethanol

**Figure 2:**
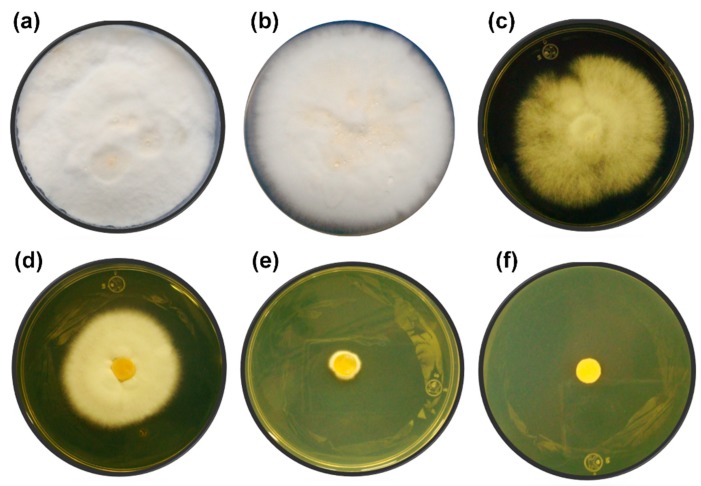
Ethanol tolerance test in solid yeast extract–malt extract agar medium (YMA) medium during10 days of incubation at 32 °C. (**a**) Control without ethanol, (**b**) 10% (*v*/*v*) ethanol, (**c**) 11% (*v*/*v*) ethanol, (**d**) 12% (*v*/*v*) ethanol, (**e**) 13% (*v*/*v*) ethanol, and (**f**) 14% (*v*/*v*) ethanol. The experiments were performed in triplicate.

During the fermentation process, the ethanol produced can inhibit the growth and viability of microorganisms by altering the cell membrane as well as the protein receptors associated with it [51,52]. Most studies related to ethanol tolerance have been carried out on yeasts. However, there is evidence of an ethanol effect on fungi. In *Phanerochaete chrysosporium*, ethanol influenced the morphology of the cell wall and led to decreased pellet diameter and fungal biomass net weight [53]. In *Trichoderma resei* cultures, the presence of ethanol (0.5%–2% *v*/*v*) hindered the secretion of cellulases, which are important enzymes for cellulose hydrolysis [53]. In Figure 2, the growth of *T. hirsuta* Bm-2 at concentrations of 0%, 10%, 11%, 12%, 13%, and 14% *v*/*v* of ethanol after 10 days of incubation is shown. *T. hirsuta* Bm-2 was able to grow vigorously at concentrations up to 10% *v*/*v*, similar to what observed without alcohol. A 50% growth could be observed at ethanol concentrations of 11% and 12% *v*/*v*, compared to the control. An incipient growth at a concentration of 13% *v*/*v* indicated that *T. hirsuta* Bm-2 was resistant to this concentration but was not able to grow adequately. At an ethanol concentration of 14%, complete growth inhibition of the fungus occurred. These results are similar to those found in the literature [22], which report the growth of the Basidiomycete *Phanerochaete chrysosporium* in the presence of ethanol concentrations of 12.2%. The level of ethanol tolerance of *T. hirsuta* obtained in this work is similar to that of *Saccharomyces cerevisiae,* which possesses a high tolerance to ethanol (concentrations up to 14%) and is traditionally used for the production of alcoholic beverages and bioethanol [54,55].

### 3.3. Ethanol Production by CBP with T. hirsuta Bm-2

Firstly, RF loads within a range of 10% to 20% (*w*/*v*) were used. It was found that at RF loads of 16%, 18%, and 20% *w*/*v*, the fungus started growing very slowly, taking up to 8 days after inoculation to cover the substrate surface. In the case of the 10% and 12% loads, the fungus did not grow adequately because of the aqueous consistency of the medium. The fungus grew adequately at 14% concentration and covered the substrate surface within 5 to 6 days after inoculation, allowing a reduction of CBP time.

**Figure 3:**
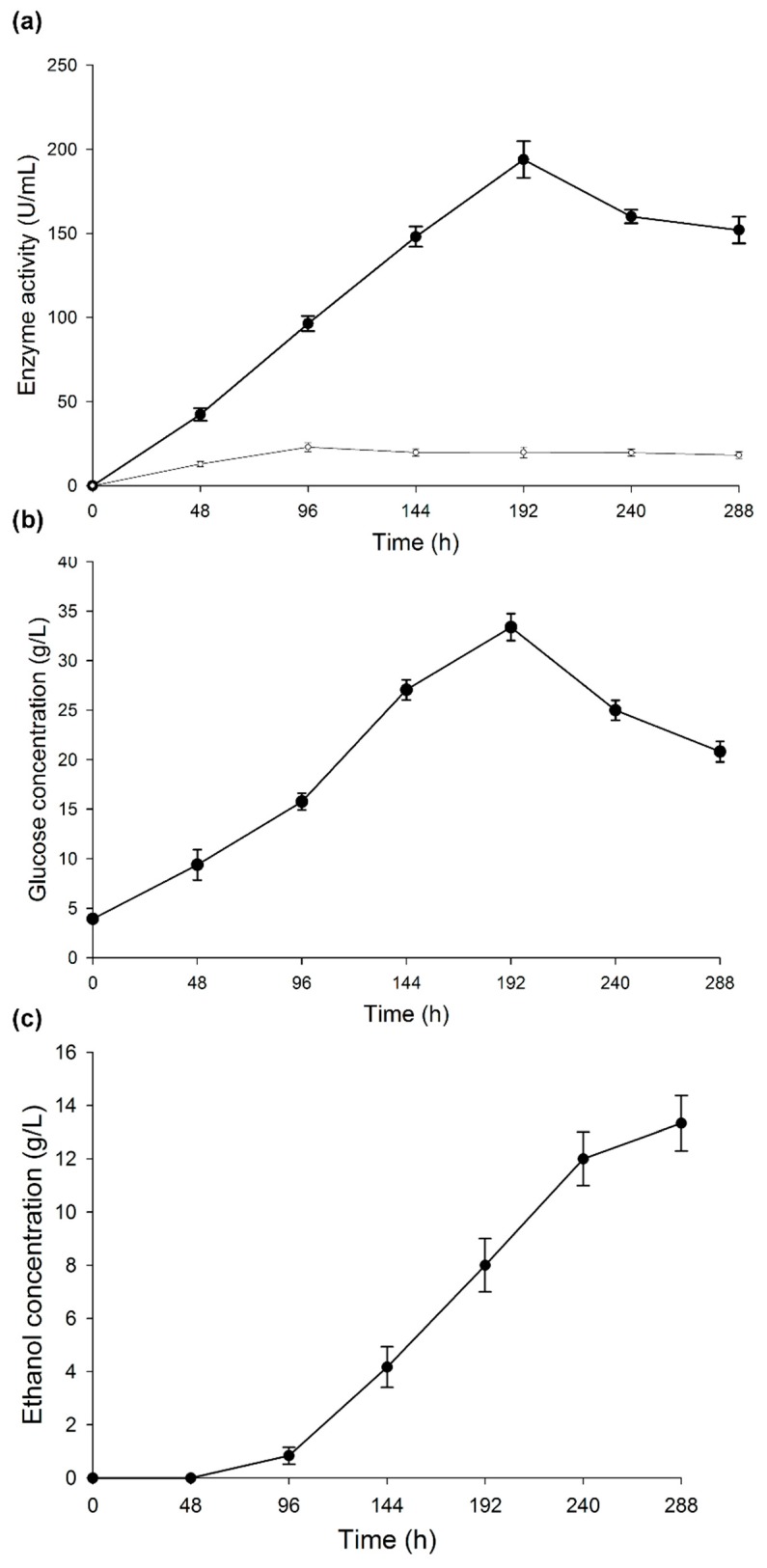
Changes during consolidated bioprocess. (**a**) α-amylase and laccase activities, (**b**) glucose concentration, and (**c**) ethanol concentration. Results are represented as the mean ± standard deviation of three parallel measurements (*n* = 3).

Figure 3a shows the profile of α-amylase activity during the development of CBP. The activity increased for 192 h, with a peak activity of 193.85 U/mL and then decreased at the end of the process. The slight decrease in the activity may be associated with an increment in ethanol concentration, since this molecule binds to the non-catalytic region of the enzyme, causing changes in its spatial configuration. However, not all enzymes are affected by ethanol in the same way. On some enzymes, such as β-glucosidase, ethanol even has a positive effect [53]. The inhibition of an enzyme by ethanol depends on other culture conditions, such as temperature and time. Sanchez et al. [44] mentioned that some authors attribute the decrease of amylase activity to a possible denaturation and/or decomposition of the enzyme due to the interaction with other components in the fermentation broth. Another factor related to a decrease in activity is starch consumption: low starch concentrations affect the induction of enzyme production, and enzymatic activity is affected by variations of the pH. However, in this case, the pH had a constant value of 5.1 during the CBP.

**Figure 4:**
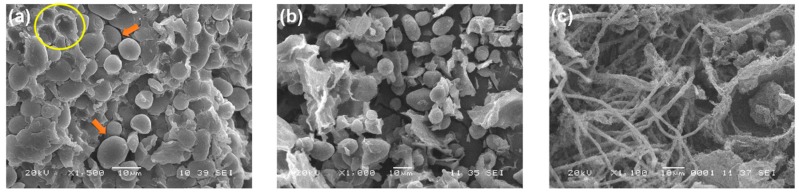
Scanning electron microscopy (SEM) of ramon flour. (**a**) without inoculation (**b**) treated with laccase extract, and (**c**) after 12 days of incubation at 32 °C.

The activity profile of the laccase enzyme can be observed in Figure 3a. After 96 h, laccase activity reached 20 U/mL and remained constant until the end of the CBP. The laccase enzyme plays an important role in the depolymerization of the fibroproteic matrix that covers the ramon starch granules (Figure 4a). To verify this, a purified laccase extract [56] was applied to a gelatinized suspension at 20% *w*/*v* (RF/distilled water) under the following conditions: 40 °C, sampling at 24 and 48 h, pH 5.1, 300 U of laccase/g of RF, 150 rpm. The results showed a fragmentation of the proteic matrix in the RF, allowing the release of the starch granules (Figure 4b). The laccase enzyme did not have any effect on the starch granules, as the concentration of free sugars in the solution at 24 and 48 h was maintained at 4 g/L. In Figure 4c, the RF at the end of the CBP is shown. The presence of mycelium indicates a vigorous growth of the fungus. Few starch granules remained at the end of the CBP, indicating a synergy between amylase and laccase enzymes. The starch granules were hydrolyzed by amylase after they were released from the flour matrix by laccase. This synchronous action of the two enzymes has recently been reported in the literature [57].

The glucose production profile (Figure 3b) was similar to that of the amylase activity. Glucose concentration was higher at 192 h (30 g/L) and then decreased to 20 g/L when amylase activity began to lower, while the fungus consumed glucose. The remaining concentration of free glucose at the end of the CBP suggests the need for implementing an additional strategy, e.g., co-cultivation of the fungus with an adequate yeast and increase in the production of ethanol.

Few natives microorganism directly convert the substrate to ethanol. In this study, we evaluated *T. hirsuta* Bm-2 in the direct production of ethanol from raw RF. Some attempts of direct bioethanol production using various microorganisms are summarized in Table 1. *S. cerevisiae* remains one of the most used microorganisms for fermentation, because of its high ethanol-producing ability, high inhibitor tolerance, and advantageous generally-regarded-as-safe (GRAS) status. *S. cerevisiae* strains lacking starch-degrading ability have been genetically engineered to express the amylase genes. However, the use of recombinant yeast strains increases the production costs as well as the biological risks, as regulations require that the genetically engineered yeasts be physically contained in order to prevent their escaping into the environment [14]. The first three examples in Table 1 refer to *S. cerevisiae* strains modified to express amylolytic enzymes, which allows them to reach high ethanol yields in a relatively short time, especially the Y294 strains, which reaches concentrations of 45 g/L in 144 h and thus offers an advantage with respect to native strains that generally require longer incubation periods. However, it should be noted that the fermentative tests on these microorganisms were performed in synthetic media with starch as a carbon source and, in the case of Y294 strains, commercial enzymes were introduced to perform hydrolysis. *S. shehatae* is a native strain that presents amylolytic enzymes production, ethanol tolerance, and fermentative capacity, which suggests to the authors that it is a suitable strain for one-step ethanol production using starch. Despite these characteristics, in this first attempt, the strain of *S. shehatae* JCM 18690 only hydrolyzed 50% of the soluble starch present after 10 days of culture, perhaps because of a low enzymatic activity or culture conditions that the authors mentioned will be resolved in later works.

Okamoto et al. [21] described the *T. hirsuta* ability to hydrolyze and ferment starch. In contrast to this work, these authors used a synthetic medium (medium T) where the carbon source was replaced by starch. In this work, instead, a complex raw material was used to obtain ethanol production, which began after 48 h of cultivation and reached a concentration of 13 g/L at 288 h (Figure 3c). On the basis of these results, a production of 123.44 mL of ethanol per kg of RF was estimated. This value is 57.95% of that reported for this substrate in previous works using SHF with a native strain isolated from the seeds of *B. alicastrum* (*C. tropicalis*) and 84.35% compared with the value reported for a standard fermentation yeast (*S. cerevisiae*) [26]. Despite the low ethanol yield, it should be taken into consideration that the CBP makes the purchase of enzymes unnecessary, because the Bm-2 strain produces laccase and amylase. Another advantage is that only one organism was used for hydrolysis and fermentation of the flour. Finally, the whole process occurred within a temperature range of 32 ± 2 °C in the same reactor, which allows a more efficient ethanol production. Similarly, no nitrogen source was added for Bm-2 strain growth, nor any enzyme inductor or buffer solution to maintain adequate pH values. Additionally, the initial pH value of the substrate ranged between 5 and 5.5, hence no initial pH adjustment was necessary, since the fungus grows naturally at these pH values. At the end of the CBP, 10% and 30% *w*/*w* of mycelium and residual flour, respectively, remained in the medium. These residues could be considered co-products to be used as animal feed, since they contain 29% and 16% of protein, respectively, giving a residual biomass with an average of 22.5% of protein. These data demonstrate that CBP using *T. hirsuta* Bm-2 with RF as a substrate is a promising strategy towards the sustainable production of bioethanol.

### 3.4. Material Flow Balance

For the CBP experiments, 13.3 g of RF (dry weight) were poured into 90.7 mL of distilled water. This mixture was sterilized, and a loss of 3.3 g was recorded. Once the mixture was cooled, 1 mL of inoculum (1.3 g) was added, initiating the CBP with a starting total weight of 102 g. After completion of the CBP, 1.33 g of mycelium and 3.99 g of non-hydrolyzed RF (dry weight) were recovered. The liquid phase was 88.43 mL and was made up of 46.03 mL of supernatant, 10.5 mL from sampling, and 31.9 mL of water calculated from humidity values of the mycelium and residual RF (85% and 83%, respectively). Also, 1.3 g of ethanol and 2 g of glucose were detected in the supernatant.

According to the biomass flow balance, there was a consumption of 68.8% of RF with respect to the initial substrate load. Table 2 shows the amounts of starch, protein, and other elements before and after the CBP. It was calculated that 5.51 g of starch (6.12 g of glucose) was consumed, which represented 67% hydrolysis of starch. From the glucose released during the process, 2.6 g was used for ethanol (calculated from the ethanol data reported above). This represents an efficiency of 28.2% with respect to the starch present in RF. In the supernatant, 2 g of free glucose was quantified, so it was calculated that the fungus, together with other RF compounds, used 1.1 g of glucose for growth.

### 3.5. Physical-Chemical Characterization

**Figure 5:**
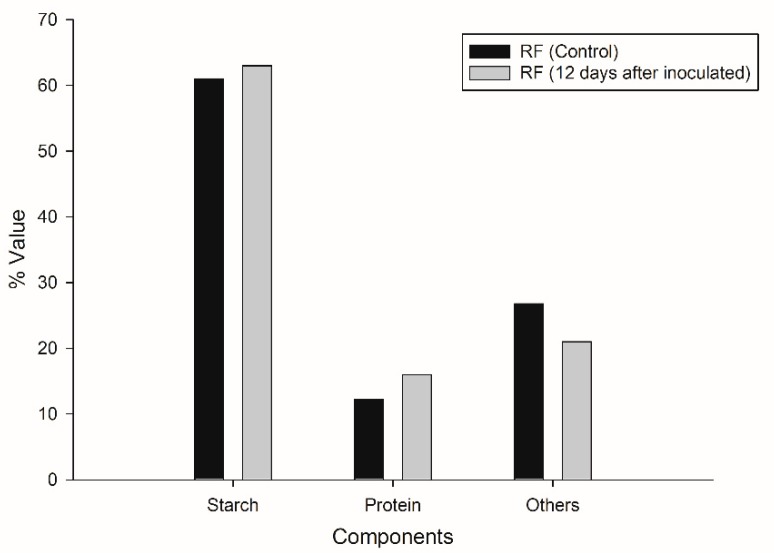
Comparative analyses of the principal components of the initial ramon flour (RF control) and the residual ramon flour (RF 12 days).

Analysis of the main components of initial and residual flour indicated that slight changes occurred in its chemical composition after CBP (Figure 5). Starch content showed a slight increase of 2 percentage points; the similarity of starch contents in the initial and the residual flour may be due to the fact that, being in stationary conditions, the enzymes were only active in the aqueous and semi-solid interface of the mixture. The protein content increased from 12.24% to 16%, which can be attributed to the fact that parts of the mycelium, with high protein content, were deposited in the flour (Figure 3c) as well as to the presence of remnants of the enzymes released by the fungus. The content decrease of the third component of RF (fibers, ashes, phenols, etc.) can be attributed to the ability of the fungus to use the phenols present in RF through the action of the laccase enzyme. Part of the insoluble fiber could also be hydrolyzed by *T. hirsuta*, as it is able to produce cellulases, in agreement with previous studies reported by Castoldi et al. [46].

These slight changes in RF composition were confirmed by elemental analysis (Table 3), where a slight increase in nitrogen content was observed in the residual RF, in accordance with the increase in protein.

**Figure 6:**
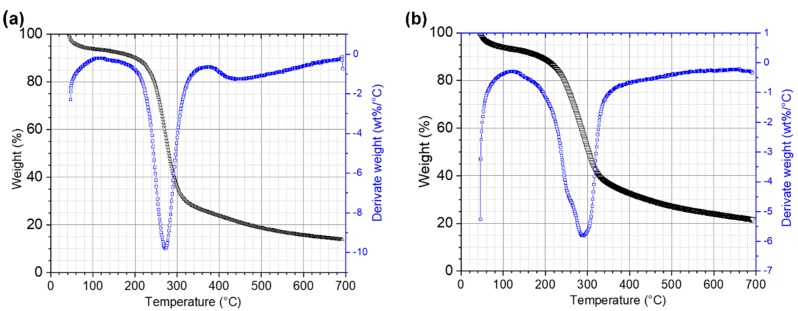
TGA and DTG curves of ramon flour. (**a**) RF control and (**b**) RF 12 days after inoculation.

TGA analysis of initial and residual RF revealed that structural changes occurred during CBP (Figure 6). In order to carry out the analysis, three stages were selected. Stage I comprised temperatures from 20 to 140 °C, at which the main events are water evaporation and volatilization of light biomass compounds. In Stage II (140 to 340 °C), decomposition of carbohydrates occurs [60]. In this stage, the presence of a double shoulder in the DTG curves of the residual RF indicates a partial enzymatic breakdown of the long amylose/amylopectin chains during the CBP. At Stage III (340 to 700 °C), substances with high molecular weight, like polysaccharides, proteins, lipids, are degraded. In this stage, the effect of the enzymes produced by the fungus that degrade the polysaccharides of the fibroprotein matrix is observed, the shoulder disappearing between 400 and 500 °C in DTG curve coinciding with that observed in scanning electron microscopy in the fragmentation of this matrix.

**Figure 7:**
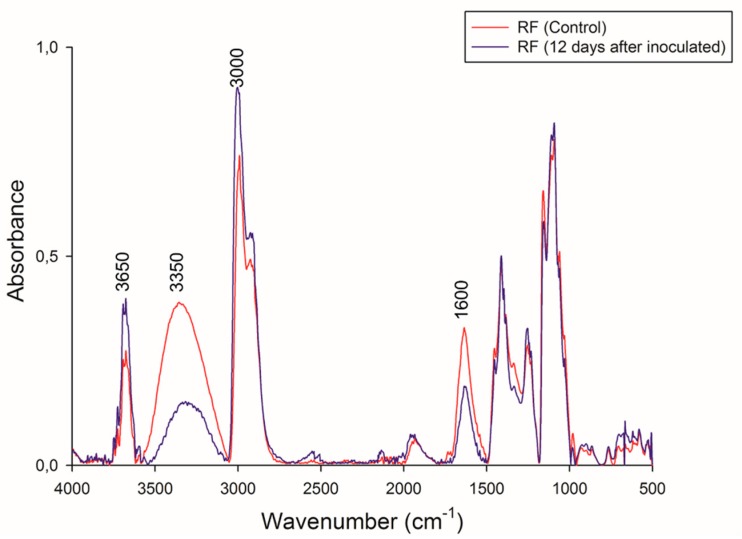
FT-IR spectral of initial and final biomass. Red line: RF control, and black line: residual RF 12 days.

FTIR spectra of RF before and after CBP are shown in Figure 7. These spectra had a similar pattern of absorption bands. The bands between 3700 and 3000 cm^−1^ can be attributed to a combination of N–H stretching vibrations from the protein and the H–O–H stretching vibrations of condensed-phase water molecules [61,62]. Specifically, the peak at 3650 cm^−1^ is associated with the N–H stretching from protein, and an increase in the intensity of this band in the residual RF can be observed. This result agrees with those of the elemental analysis mentioned above. 

Both spectra showed a broad peak in the OH stretching region at 3350 cm^−1^. This band is associated with the hydroxyl groups of insoluble fiber present in the RF. In the residual RF spectra, a decrease in the intensity of this band indicates the degradation of compounds that possess –OH moieties (mainly starch, phenols, and cellulose). 

The differences in band intensities at 2800 and 3000 cm^−1^ can be attributed to variations in the amounts of amylose and amylopectin [63] and agree with the TGA analysis mentioned above. The reduction of band intensities may be associated with the lower exposure of the –CH groups of amylose and amylopectin at the beginning of CBP due to RF compaction.

A decrease at 1600 cm^−1^ was also observed. Different groups can be associated with this band, e.g., amides, carboxyl groups, C=C aromatics, lignin, and amino acids [64,65].

## 4. Conclusions

The results obtained in this work revealed that the native Bm-2 strain has the ability to simultaneously produce α-amylase and laccase enzymes whit activities of 135 and 40 U/mL, respectively, which are involved in the deconstruction of the polymers present in the ramon flour. This feature allows the use of this fungus in a consolidated bioprocess for ethanol production from *B. alicastrum* seed flour at a concentration of 13 g/L. On this basis, 123.4 mL of pure ethanol could be produced from 1 kg of flour. The consolidated bioprocess developed in this work does not require the addition of commercial enzymes to hydrolyze the starchy material nor of a nitrogen source during the fermentation stage. In addition to these qualities, the residual biomass, with average protein content of 22.5%, could improve the economic viability of the overall process in terms of the biorefinery concept.

## Figures and Tables

**Table 1:** Ethanol production through consolidated bioprocessing (CBP).

Substrate	Microorganism	Ethanol [g/L]	Referencia
Corn starch	*Saccharomyces cerevisiae* Mnua1	9.03 after 240 d	[58]
Raw starch and glucose	*S. cerevisiae* Y294[AteA-GlaA]	45.77 after 144 h	[59]
Raw starch and glucosa	*S. cerevisiae* Y294[GlaA-AteA]	45.85 after 144 h	[59]
Soluble starch	*Scheffersomyces shehatae* JCM 18690	9.2 after 240 h	[14]
Starch	*Trametes hirsuta*	9.1 after 96 h	[21]
Raw Ramon Flour	*T. hirsuta* Bm-2	13 after 288 h	This work

**Table 2:** Consumption analysis of the main components of ramon flour during CBP.

Component	RF (Control) g	RF (12 Days after Inoculated) g	% Material Consumed
Starch	8.11 ± 0.67	2.60 ± 0.35	67
Protein	1.63 ± 0.44	0.66 ± 0.50	59
Others	3.56 ± 0.50	0.88 ± 0.34	75

**Table 3:** CHNS elemental analyses of the initial ramon flour and residual ramon flour.

Sample	N (wt %)	C (wt %)	H (wt %)
Initial RF	2.04 ± 0.10	41.22 ± 0.80	6.41 ± 0.01
Residual RF	2.79 ± 0.18	41.87 ± 0.16	6.10 ± 0.04
